# Associations between Post-Intensive Care Syndrome Domains in Cardiac Arrest Survivors and Their Families One Month Post-Event

**DOI:** 10.3390/jcm13175266

**Published:** 2024-09-05

**Authors:** Danielle A. Rojas, George E. Sayde, Jason S. Vega, Isabella M. Tincher, Mina Yuan, Kristin Flanary, Jeffrey L. Birk, Sachin Agarwal

**Affiliations:** 1 Department of Neurology, Columbia University Irving Medical Center, New York, NY 10032, USA; 2 Department of Psychiatry, Columbia University Irving Medical Center, New York, NY 10032, USA; 3 Vagelos College of Physicians and Surgeons, Columbia University, New York, NY 10032, USA; 4 Cardiac Arrest Family Member Stakeholder & Advocate, Glaucomflecken LLC, Eugene, OR 97401, USA; 5 Center for Behavioral Cardiovascular Health, Columbia University Irving Medical Center, New York, NY 10032, USA

**Keywords:** cardiac arrest, post-traumatic stress disorder, cognition, patient and family dyads, post-intensive care syndrome

## Abstract

**Background**: Post-intensive care syndrome (PICS) affects many critical care survivors and family members. Nevertheless, the relationship between PICS-relevant domains in cardiac arrest (CA) survivors and psychological distress in their family members (henceforth, PICS-F) remains underexplored. **Methods**: We enrolled consecutive CA patients admitted between 16 August 2021 and 28 June 2023 to an academic medical center, along with their close family members, in prospective studies. Survivors’ PICS domains were: physical dependence (Physical Self-Maintenance Scale, PSMS), cognitive impairments (Modified Telephone Interview for Cognitive Status, TICS-M), and post-traumatic stress disorder (PTSS) symptoms (PTSD Checklist—PCL 5), as well as PICS-F (PCL-5 Total Score). Hierarchical multivariate linear regressions examined associations between PICS-F and survivors’ PICS domains. **Results**: Of 74 dyads (*n* = 148), survivors had a mean (*SD*) age of 56 ± 16 years, with 61% being male and with a median hospital stay of 28 days. Family members (43% spouses) were slightly younger (52 ± 14 years), predominantly female (72%), and of minority race/ethnicity (62%). A high prevalence of PICS assessed 28.5 days (interquartile range 10–63) post-CA was observed in survivors (78% physical dependence, 54% cognitive impairment, 30% PTSS) and in family members (30% PTSS). Survivor PTSS was significantly associated with family member distress (*β* = 0.3, *p* = 0.02), independent of physical dependence (*β* = 0.0, *p* = 0.9), cognitive impairment (*β* = −0.1, *p* = 0.5), family member characteristics, and duration of hospitalization. **Conclusions**: Both CA survivors and their family members showed substantial evidence of likely PICS. Survivor PTSS is notably associated with family member distress, highlighting the need for dyadic interventions to enhance psychosocial outcomes.

## 1. Introduction

Advancements in intensive care medicine have increased the likelihood of survival for critically ill patients [1,2]. However, more than half of intensive care unit (ICU) survivors face a range of new or worsening physical, cognitive, and psychological difficulties collectively known as “post-intensive care syndrome” (PICS) [3,4]. This syndrome not only affects the survivors’ long-term quality of life [5] but also presents significant challenges for their families, termed post-intensive care syndrome in family members (PICS-F) [2,6]. It encompasses psychological distress, physical challenges, and socio-economical burdens that begin with the emotional impact experienced by the family when the patient is admitted to the ICU [1,2,7]. Psychological impairments have significant implications for a family’s ability to support the recovery of ICU survivors [8,9].

Evidence suggests that family members of ICU survivors experience higher levels of post-traumatic stress symptoms (PTSS) compared to the patients themselves at three months post-discharge [10]. A similar trend is observed among family members of cardiac arrest (CA) survivors, where PTSS prevalence is notably higher among family caregivers (35–50%) compared to the survivors themselves (30%) at discharge [11,12,13,14,15]. This may be due to the unique experience associated with CA for both survivors and their close family members. Unlike other ICU conditions, survivors of CA face the psychological impact of having been “clinically dead” and then revived, with their family members often thrust into roles of emergency responders and caregivers with little preparation [16,17].

Existing research has focused primarily on identifying the most common deficits and how troubling they are to CA survivors and their families [12,13,14,18,19,20,21,22]. Few [23,24,25] have examined patients and their caregivers together simultaneously (e.g., dyads), which is important as literature from other medical illnesses clearly shows that both psychological resilience and distress following illness are significantly interrelated within dyads [26,27,28]. Qualitative research [13,14,29,30] identifies emotional disturbances as a significant challenge for family members of CA survivors, often due to feelings of helplessness in restoring normalcy for both themselves and their loved ones. Emerging evidence [31] and theory [32] suggest a bidirectional impact of severe stress between patients and their family members, indicating that emotional states may transfer between individuals.

The severity of the illness [33], including prolonged ICU stay [34], has been associated with PICS-F, but it is unclear which PICS domains have a significant and independent relationship with PICS-F in the acute period of critical illness. To fill this knowledge gap, we conducted a cross-sectional study of a longitudinal cohort examining the association between PICS domains—cognitive, psychological, and physical—in CA survivors, and the psychological distress experienced by their family members approximately one month after the event. We hypothesized that the survivors’ emotional distress would independently and significantly correlate with family member distress. Understanding these dynamics is crucial for preventing disruptions in the survivor–family relationship [35] and for developing targeted interventions that enhance recovery for both survivors and their families.

## 2. Methods

### 2.1. Study Population

Participants, i.e., CA survivors and their close family members, were enrolled from the daily screening logs of an NIH/NHLBI-funded research study (R01-HL153311; R01-HL151850) recruiting consecutive CA patients admitted between 16 August 2021 and 28 June 2023 to any of the eight intensive care units at the Columbia University Irving Medical Center, New York. The center serves the Washington Heights area of Northern Manhattan, a high-risk, low-resource population comprising 68% Hispanic ethnicity, 20% non-Hispanic White, 33% with limited English, and 20% below the Federal poverty line [36]. The study protocol was approved by the Columbia University Institutional Review Board. This study adhered to the STROBE guidelines (see Supplementary Methods).

### 2.2. CA Survivors

The main inclusion criteria included current hospitalization with either in-hospital or out-of-hospital CA defined as the provision of cardiopulmonary resuscitations. Exclusion criteria were severe brain injury defined as a modified Rankin Scale > 4 [37] or death within 30 days of the CA, speaking neither English nor Spanish, unavailability for follow-up, or inability to complete study procedures due to unreliable phone or internet access. Additional exclusion criteria were the absence of close family members or the inability to visit during working hours.

### 2.3. Close Family Members

The study protocol required a waiting period of 48 h post-arrest before the approach was attempted by the study team. This approach was implemented to avoid placing unnecessary emotional strain on the families, and to ensure that enrolled close family members were of CA patients who would survive at least 30 days or until hospital discharge. Typically, death rates are higher within the first 48 h following a cardiac arrest.

To be classified as a “close family member”, participants were required to be either the patient’s designated healthcare proxy or someone with a close, immediate relationship to the patient who was present at the bedside during the consent process. Simply being present at the bedside was not sufficient; in cases where multiple individuals were present, they were asked to specify who the patient’s primary contact or caregiver was. This procedure helped identify those individuals who were most closely connected to the patient and most affected psychologically by the traumatic event.

### 2.4. Procedure

Both survivors and their family members completed assessments either via telephone or in person, in case the survivor was still hospitalized.

### 2.5. PICS Measures

#### 2.5.1. Physical Dependence

The Physical Self-Maintenance Scale (PSMS) covers six domains: toilet, feeding, dressing, grooming, physical ambulation, and bathing. A scoring system was used where a score of 1 was assigned only if the highest level of function was achieved in each category, otherwise, the score was 0. The total summed score, ranging from 0 to 6, reflected the survivor’s functional performance, with higher scores indicating a higher functional level. The PSMS is a validated scale and showed a correlation of 0.62 with a physician’s rating of functional health and 0.61 with an Instrument of Activity of Daily Life scale [38]. An inter-rater reliability of 0.91 and Guttman reproducibility coefficient of 0.96 were reported [38].

#### 2.5.2. Cognitive Impairment

The modified Telephone Interview for Cognitive Status (TICS-M), a valid [39,40], commonly used global cognitive screening test, captured four domains: orientation, language/attention, verbal episodic memory, and semantic memory [40,41]. This performance-based measure, which has scoring adjusted for educational attainment [42], ranges from 0 to 50 points. Higher scores indicate better cognitive function and scores below 33 indicate cognitive impairment [43].

#### 2.5.3. PTSS in Survivors and Psychological Distress in Families (PICS-F)

The Post-traumatic Stress Disorder (PTSD) Checklist (PCL-5) is an extensively validated, 20-item scale developed by the National Center for PTSD that corresponds to DSM-5 criteria for PTSD. The PCL-5 has been validated for telephone administration [44] and performed well for our CA participants [45]. The PCL-5 queries PTSD symptomatology in relation to an identified stressful experience in the past month. The PCL-5 has adequate test–retest reliability and excellent sensitivity/specificity for PTSD clinical diagnosis prediction [46].

In this study, the research coordinator conducted a structured interview with each survivor; the PCL-5 items were anchored toward the acute CA event and subsequent hospitalization. Each structured interview began with the co-ordinator reading, “The event you or your loved one experienced was a CA on (date). I will read a list of problems and complaints that people sometimes have in response to stressful experiences. Please listen carefully, then indicate on a scale of 1–5 how much you have been bothered by this event and subsequent hospitalization in the past month. The scale of 1–5 is as follows: 1 is ‘not at all’, 2 ‘a little bit’, 3 ‘moderately’, 4 ‘quite a bit’, and 5 ‘extremely’”. In addition to the co-ordinator reading what each number corresponded to, a reference card was given to the participants who had the five choices presented to them. This was done so they could refer to all five responses during the interview. The PCL-5 cut-point score ≥ 31 is indicative of probable PTSD across samples [47].

The PTSS subscales matched the four symptom clusters for PTSD within DSM-5: re-experiencing (criterion B, items 1–5, max score = 20), avoidance (criterion C, items 6–7, max score = 8), negative alterations in cognition and mood (criterion D, items 8–14, max score = 28), and hyperarousal (criterion E, items 15–20, max score = 24).

### 2.6. Bio-Medical Data

Medical variables were extracted from electronic health records, including insurance classification, location of the CA, cause of the CA, initial rhythm, time of return of spontaneous circulation (ROSC), hospital length of stay, comorbid medical conditions using the Charlson Index [48], and discharge disposition.

### 2.7. Selection of Covariates and Models

We followed the recommendation that covariates be selected for inclusion a priori [15]. Based on published findings of factors that might confound the association between PICS domains and PICS-F, prolonged hospital stay [34,49] as a marker of severity of illness, and family attributes associated with increased risk of PICS-F, consistently included younger age [50,51,52,53], female sex [33,34,51,54,55], educational attainment [56,57], and spouse/partner as relationship status [51,58].

To explore other independent associations between survivors’ potential PICS domains and families’ symptoms of distress, univariate analyses, as well as family psychological distress as a dichotomous variable (PCL-5 ≥ 31), were performed with several items. The factors considered were among (1) family member attributes (e.g., socio-demographic variables including race/ethnicity, preferred language, employment status, marital status, relation to survivor, witnessing the CA), (2) survivor-related attributes (e.g., age, sex, sexuality, race/ethnicity, preferred language, educational attainment, income, insurance classification, location of the CA, Charlson Comorbidity Index), (3) illness-specific characteristics (i.e., cause of the CA, initial rhythm, time to ROSC), and (4) hospital-related outcomes (i.e., discharge functional status, disposition).

### 2.8. Statistical Analysis

Continuous variables were summarized as mean (*SD*) or median (interquartile range), while categorical variables were presented as frequency (%). Fisher’s exact test and the Wilcoxon-rank sum test were used to compare participants in each group. A kernel density estimate (KDE) plot was created to establish the normality of family members’ distress scores (Supplementary Figure S1). We used hierarchical linear regressions to analyze the associations between PICS-F and PICS domains. In Model 1, we adjusted for PICS domains (PSMS total score, TICS-M total score); Model 2 included family attributes (i.e., age, female sex, higher level of education, spouse/partner or not); Model 3 incorporated hospital length of stay, reflecting the severity of illness and duration of exposure. Both unstandardized (B) with 95% confidence intervals and standardized (*β*) beta coefficients were estimated. Our models fulfilled assumptions for linear regressions. The variables did not present with multicollinearity; the variance inflation factors of the multivariate models were <2. To ensure the robustness of our regression model, we employed both the Linktest and the Ovtest. The Linktest assessed potential specification errors by examining the significance of the squared predicted values. The test revealed a coefficient of −0.01 with a standard error of 0.02 for the squared predicted values, with a *p*-value of 0.4 indicating that the model specification was well specified. Additionally, the Ovtest, which evaluates whether higher-order terms of the fitted values improve the model, yielded an F-statistic of 2.2 and a *p*-value of 0.1 This result supports the adequacy of our model specification. An alpha threshold of 0.05 was applied, and all tests were two-tailed. We then used Spearman’s correlation to create a matrix for family members’ and survivors’ PCL-5 total and subscales, with positive rho values > 0.5 demonstrating a moderate-to-strong correlation [59].

### 2.9. Sample Size and Power Analysis

The finding that the survivors’ PTSS was significantly associated with the families’ psychological distress in the fully adjusted model suggests that limited power was not an issue. Nevertheless, we performed post hoc power analysis using an *R*-squared test in a multiple linear regression to test the significance of all coefficients included in the final model. With alpha = 0.05, *N* = 74, *R*-squared of 0.21, and 8 tested covariates, the estimated power was 87%. All statistical analyses were performed using STATA 18.

## 3. Results

### 3.1. Participants

Of the 684 patients admitted with CA, 229 met eligibility criteria and 161 of those were enrolled. Among enrolled survivors of CA, 45 family members met the exclusion criteria, and 116 family members were approached. After 20 family members declined due to lack of interest or being overwhelmed with emotional or logistical burdens, 96 were enrolled. With 22 families either lost to follow-up (*n* = 20) or having withdrawn from the study (*n* = 2), 74 survivors and their corresponding 74 close family members with complete 1-month assessments were included in the primary analysis (Supplementary Figure S2: CONSORT diagram).

### 3.2. Survivor Characteristics

The average age of survivors was 56 ± 16 years. The majority identified as male (61%) and heterosexual (99%). The sample was racially and ethnically diverse; only 46% reported their race as Non-Hispanic White, 16% as Black, and 37% reported Hispanic/Latino ethnicity. More than half (55%) attended at least trade school or college, a large proportion were either uninsured or on Medicaid (44%), and 47% had an average annual income of less than USD 60,000, slightly lower than the median household income of USD 69,717 [36]. While 66% of survivors had poor functional status (modified Rankin Scale score > 2), more than half (57%) were discharged home versus an inpatient acute or subacute rehabilitation facility (Table 1).

Most patients had an in-hospital arrest (73%). The cause of the arrest was from a cardiac etiology in 47% of cases, and non-shockable rhythms were the initial rhythm in more than half of cases (59%). While the median time to ROSC was 5 min, the median hospital length of stay was 28 days, and survivors had significant co-morbidities (median Charlson Comorbidity Index of 3).

### 3.3. Family Member Characteristics

The average age of family members was 52 ± 14 years. The majority identified as female (72%) and from a minority racial/ethnic background (62%), with Hispanic/Latino (51%) being the most represented group. More than half of the family members had college degrees or higher (51%) and worked full- or part-time before hospitalization (55%). The most common relationship to the survivor was spouse/partner (43%), followed by adult child (23%). Only 18 (24%) family members witnessed their loved one’s CA (Table 2).

### 3.4. Prevalence of Survivor and Family PICS-Related Domains

**Figure 1:**
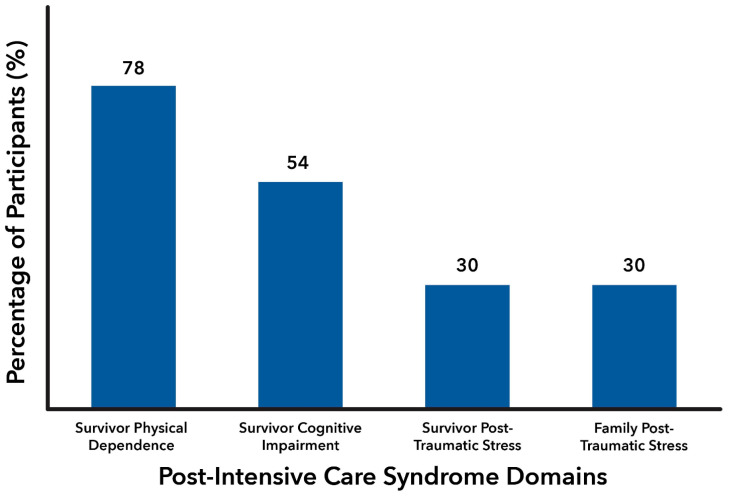
Prevalence of post-intensive care syndrome domains in family members and survivors at 1 month after cardiac arrest.

In assessments conducted at a median duration of 28.5 (interquartile range 10–63) days after cardiac arrest, three out of four survivors reported dependency on at least one of the key domains of activities of daily living (78%), with more than half (54%) experiencing global cognitive dysfunction. Approximately one out of three survivors (30%) screened was positive for PTSS one month after CA (Figure 1). Notably, the overall prevalence of psychological distress in close family members was also 30%.

### 3.5. Associations between PICS Domains in Survivors’ and Family’s Psychological Distress

The univariate regression analysis revealed a significant positive association between survivor PTSS and family member psychological distress scores (*β* = 0.4, 95% CI [0.2, 0.6], *p* < 0.01). No statistical significance was found for the other two PICS domains—PSMS (*β* 551= 0.0, *p* = 0.8) and TICS-M (*β* = −0.1, *p* = 0.3) (Table 3).

In a multivariable analysis, survivors’ PTSS remained consistently and significantly associated with family members’ distress scores across all three models. Model 1 adjusted for survivor’s PSMS and TICS-M total scores (*β* = 0.4, 95% CI [0.1, 0.6], *p* < 0.01), Model 2 additionally adjusted for family members’ age, sex, education, and partner/spouse as relationship (*β* = 0.3, 95% CI [0.1, 0.6], *p* = 0.02), and finally Model 3 included hospital length of stay (*β* = 0.3, 95% CI [0.1, 0.6], *p* = 0.02; medium-to-large effect size, η^2^ = 0.1, 95% CI [0.002, 0.2]).

### 3.6. Correlation Matrix for Family Member and Survivor PCL-5 Total and Subscales

While significant correlations were seen between total and subscales of PCL-5 for both survivor and family members, the survivors’ negative alterations in cognition and mood (Criterion D) showed a moderate-to-strong positive correlation with the family members’ PCL-5 total score (*ρ* = 0.5, *p* < 0.001) (Table 4).

### 3.7. Other Significant Factors Shown to Have Association with Family’s Psychological Distress

There was a higher prevalence of psychological distress seen in family members of Hispanic/Latino ethnicity compared to non-Hispanic whites (77% vs. 39%, *p* < 0.01). Furthermore, discharge disposition to home vs. an inpatient rehab facility also showed a significant difference in the proportions of family members screening positive for psychological distress (82% vs. 45%, *p* < 0.01) (Supplementary Table S1). In follow-up testing, the means of the family’s continuous PCL-5 scores differed for the two dispositions (Home: 27 ± 20 vs. Facility: 14 ± 11, *p* < 0.01). These findings were further confirmed by the significant univariate associations of family ethnic status and survivor’s discharge dispositions seen with family members’ psychological distress (Supplementary Table S2).

## 4. Discussion

This study underscores the significant impact that ICU survivors’ PTSS likely has on the mental health of their close family members [60]. Our principal observation is that, among the three primary domains related to PICS—physical, cognitive, and psychological—the severity of PTSS in survivors was significantly and independently associated with the psychological distress experienced by family members. This association persisted even after accounting for the other two domains and known risk factors associated with PICS-F, such as younger age, female sex, lower educational level, intimate relationship status, and longer hospital length of stay.

Psychological distress is a key feature of PICS affecting both survivors and their families following critical illness and hospitalization. Our study revealed that close to one-third of both survivors and their family members screened positive for post-traumatic stress. This is in accordance with previous research from various independent prospective cohorts of CA survivors [12,13,18,24]. The reported prevalence rates among close family members are mostly derived from registry data and range from 35% to 50% [14].

Interestingly, the study found no significant associations between family distress and the survivors’ level of physical dependency or cognitive impairment. In our previous studies with survivors, cross-sectional analyses conducted at hospital discharge and six months post-discharge revealed that PTSS was a strong, independent predictor of perceived overall non-recovery, irrespective of cognitive impairment or significant physical dependency [61,62]. These results contribute to the ongoing conversation about how and how much the characteristics and recovery levels of survivors have an impact on the stress experience of family members. The existing literature on stroke survivors indicates that both mental health and cognitive function [63,64,65], as well as physical impairment [66,67,68], are associated with higher levels of family caregiver stress. However, there is limited research in CA or similar critical care populations, precluding any meaningful comparisons.

Emerging evidence underscores the bidirectional and dynamic nature of psychological distress between patients and family members following ICU stays [31,69]. This emphasizes the interdependence in family caregiving relationships and the interpersonal nature of stress within this context [27]. A dyadic analysis is likely to reveal the true interaction between family members’ and CA survivors’ mental health within their relationship. Understanding these dynamics is crucial for identifying family members who are at risk of adverse outcomes and for developing interventions to support them in managing these challenges. 

Interestingly, survivors’ demographic and clinical characteristics, such as age, sex, illness severity indicators (including hospital length of stay), and functional status after ICU discharge, did not correlate with the level of psychological distress experienced by family members. This observation is consistent with other studies that have failed to find a reliable association between these factors and family distress [33,70,71]. Regardless of the illness severity, the PTSS experienced by survivors remains a significant challenge for their family members [26]. Our study did not include family members of patients who died during hospitalization—likely those most severely ill and experiencing the highest levels of distress—to differentiate between complicated grief and psychological distress in families related to ICU admissions. 

Notably, in our sample, the levels of psychological distress among family members varied depending on the family’s ethnicity, education level, and eventual discharge disposition of their loved one. All three factors could be interrelated and underscore the potentially related issues of reduced access to resources and a limited ability to comprehend the information provided by the medical team regarding CA [8,13]. In the institutional protocol where this study was conducted, all survivors of cardiac arrest were evaluated by physical, occupational, and speech therapists before hospital discharge to determine their dispositions. Despite this, insurance and patient–family cultural preferences between inpatient and outpatient rehabilitation services may have played a role in determining the final disposition decision. This may explain the discrepancy between survivors with high physical dependency on their activities of daily living and low receipt of inpatient rehabilitation. Our study findings contrast with previous study’s results in which family members of survivors discharged to institutional settings after acute care hospitalization experienced significantly higher levels of depression at two months post-discharge compared to those whose loved ones were discharged home [72,73]. In contrast, our study observed increased psychological distress among family members when survivors were discharged directly home. This discrepancy may be attributed to differences in the specific outcomes measured and the timing of assessments. The lack of formal guidelines for discharge planning, along with limited informational and psychological support for family members of CA survivors, may contribute to the heightened distress associated with home discharge. Discharge to a rehabilitation facility might mitigate some of the uncertainties related to home discharge and offer a sense of continuity in medical care. Since examining the impact of discharge dispositions on family members’ perceptions and distress levels was not the primary aim of this study, further research is needed to explore these aspects more comprehensively. 

Compared to other PTSS clusters (i.e., re-experiencing, avoidance, and arousal), survivors’ symptoms of negative cognition and mood were significantly and strongly correlated with family member distress. This cluster is characterized by negative thoughts about oneself, other people, or the world, hopelessness about the future, memory problems, including not remembering important aspects of the traumatic event, difficulty maintaining close relationships, feeling detached from family and friends, lack of interest in activities previously enjoyed, difficulty experiencing positive emotions, and feeling emotionally numb [74]. Unlike a stroke or a heart attack, the absence of memories around the event is unique in this population and is the most reported concern following CA [24,75]. On the contrary, family members who witnessed the CA are often plagued by memories of the event and fear of recurrence [76]. It has been hypothesized that family members might be unable to discuss the event with the survivor because of the lack of shared memory, which results in misunderstandings and leads to psychological distress in the family member [77].

### 4.1. Strengths

Our study has several notable strengths. To our knowledge, this study has the largest sample of CA survivor–family dyads recruited prospectively from a hospital setting to date. These findings help fill a gap in the literature which predominantly features CA survivor outcomes with less of a focus on family members. Our sample includes a diverse group of family members, a wide race/ethnicity distribution, and both in-hospital and out-of-hospital CAs. This speaks to the high generalizability of our findings.

### 4.2. Limitations

One limitation of this study is that we did not have information on family members’ psychological distress assessments prior to their relatives’ hospitalization. We could not determine whether family members were already experiencing psychological distress before hospitalization or how much of their distress may have been exacerbated by the ICU experience and the subsequent recovery process. Since critical illness often occurs unexpectedly, it is challenging to obtain baseline mental health data for family members. Despite this, the prevalence of distress reported in this study is significantly higher than that observed in the general population, and our assessments were specifically aligned with the CA and the following hospitalization. 

Second, loss to follow-up affected approximately 23% of the families, reducing the expected sample size. This loss could potentially impact the robustness of our findings and should be acknowledged as a limitation. Although this concern is somewhat mitigated by the lack of significant differences between the analyzed group and those lost to follow-up (see Supplementary Table S3), we cannot definitively rule out the possibility that family members with severe psychological distress were among those who either declined participation or were lost to follow-up.

The study aimed to estimate psychological distress or post-traumatic stress symptoms (PTSS) in family members, with the primary outcome assessed at the earliest recommended time by the DSM-5, which is 1 month [47]. However, it is important to note that several patients (45%, *n* = 34) were still hospitalized when their disability assessments were conducted. This may have influenced the assessment outcomes and should be considered when interpreting the results.

Previous research, including studies on strokes [78], has shown that psychological symptoms in family members tend to be persistent and are most accurately predicted by their psychological state during the acute phase. Our own research further supports this, indicating that survivors’ PTSS during the acute period was associated with a three-fold increased risk of major cardiovascular events [45]. This underscores the importance of evaluating family members’ psychological states early in the post-cardiac arrest period and considering both patient- and family-related factors. To better understand the long-term durability of these associations, a follow-up study extending beyond 1 month would be ideal.

In terms of model selection, the consistency between the significant unadjusted and the fully adjusted association between the survivors’ PTSS and family members’ psychological distress scores further supports our decision to control for theoretically important covariates. Concerns about model overfitting led us not to model other variables that may influence this association (e.g., survivors’ comorbidities [33], lower socioeconomic status [51,79,80], living alone [50,81], and witnessing cardiopulmonary resuscitation) [82]. However, these factors were non-significant in univariate analysis, which diminished our concern about omitting them from the models.

In this study, we observed a significant association between cardiac arrest survivor PTSS and psychological distress in close family members. However, this study is observational and does not provide any evidence of causality. While our analysis controlled for several confounding factors, residual confounding may still influence the observed relationship. Alternative explanations, such as the family’s potential lack of awareness regarding how the survivor’s physical dependence and cognitive impairment might impact their return to work or societal participation over time, were considered. However, the observational nature of our study limits our ability to establish causality. Future research should utilize experimental designs or randomized controlled trials to validate these findings and explore underlying mechanisms.

### 4.3. Future Directions

This study represents one of the first steps in exploring the relationship between the type of deficits experienced by the survivors after CA and the family’s emotional experience. Future studies should also capture certain protective factors that have been associated with family distress. High resilience could influence survivor and family perceptions of disease recovery [56], their perceptions around available social support, and the state of family dynamics [83] are a few known so far. Other ICU-related factors, such as unsatisfactory patient-provider communication in ICU [49,55] and insufficient information about the disease, prognosis, and treatment [84,85], also warrant a separate study and are easily modifiable targets for intervention by ICU staff, if demonstrated to be significant.

Time may also serve as a moderating factor and should be considered in research that looks at outcomes beyond the acute period. Little research has been reported on the stress levels of CA survivors and family member dyads over time. We know that nearly 50% of family members of CA survivors report persistent distress at one year [21,60].

## 5. Conclusions

CA survivors’ post-traumatic stress symptoms within the initial weeks after CA, and not levels of physical disability or cognitive impairment, may represent a meaningful risk factor for psychological distress in family members. These findings may help to improve prevention strategies concerning PICS-F by indicating the need to assess survivors’ and family members’ characteristics and demographics early on during their ICU stay and, consequently, allowing for the early identification of at-risk individuals and the timely implementation of adequate support services.

## Figures and Tables

**Table 1:** Survivor characteristics.

	Patients (*N* = 74)
Age, years, mean ± SD	56 ± 16
Female sex	39 (29)
Heterosexual	99 (73)
Race/ethnicity	
Non-Hispanic White	46 (34)
Black	16 (12)
Hispanic/Latino	37 (27)
Other	1 (1)
Language	
English	82 (61)
Spanish	18 (13)
Educational attainment	
High school diploma/GED or less	45 (34)
Trade school/some college or more	55 (41)
Income	
Less than USD 59,999	47 (35)
USD 60,000–89,999	8 (6)
Greater than USD 90,000	27 (20)
Unknown	19 (14)
Insurance	
Uninsured	1 (1)
Insured (Medicaid)	43 (32)
Insured (Medicare)	28 (20)
Insured (Private)	28 (20)
How survivor first learned about cardiac arrest	
Family member	39 (29)
Clinical staff	28 (28)
Research staff	5 (4)
Self-remembrance	18 (13)
Cause of cardiac arrest	
Cardiac etiology	47 (35)
Respiratory etiology	24 (18)
Circulatory etiology (i.e., shock)	23 (17)
Other	4 (3)
Unknown	2 (2)
Cardiac arrest site	
Home	27 (20)
In-hospital	73 (55)
Initial rhythm	
Shockable rhythm (Ventricular Tachycardia/Fibrillation)	33 (25)
Non-shockable rhythm (Asystole/Pulseless electrical activity)	59 (44)
Unknown	8 (6)
Time to ROSC, minutes, median (interquartile Range)	5 (3–10)
Hospital length of stay, days, median (interquartile Range)	28 (16–42)
Charlson Comorbidity Index, median (interquartile Range)	3 (1–5)
Disposition	
Home	57 (41)
Inpatient acute rehabilitation	39 (28)
Inpatient subacute rehabilitation/skilled nursing	4 (3)
Poor functional status, modified Rankin Scale Score > 2	66 (49)
Physical dependence (PSMS), mean ± SD	3 ± 2
Cognitive function (TICS-M), mean ± SD	31 ± 7
Psychological distress (PCL-5), mean ± SD	23 ± 17

Note. Values are % (*n*) unless otherwise reported. PCL-5 = Post-Traumatic Stress Disorder Checklist for DSM-5. PSMS = Physical Self-Maintenance Scale. ROSC = Return to Spontaneous Circulation. TICS-M = Modified Telephone Interview for Cognitive Status.

**Table 2:** Family member characteristics.

	Family Members (*N* = 74)
Age, years, mean ± SD	52 ± 14
Female sex	72 (54)
Race/ethnicity	
Non-Hispanic White	32 (24)
Black	11 (8)
Hispanic/Latino	51 (38)
Other	6 (5)
Language	
English	84 (63)
Spanish	16 (12)
Educational attainment	
8th grade or less	1 (1)
Some high school	8 (6)
High school diploma or GED	10 (7)
Trade school/vocational school	8 (6)
Some college, no degree	22 (16)
College degree	30 (22)
Some graduate school, no degree	4 (3)
Graduate degree	17 (12)
Employment status	
Employed, full-time	43 (32)
Employed, part-time	12 (9)
Disabled, permanently or temporarily	6 (4)
Temporarily laid off, sick leave, or maternity leave	3 (2)
Homemaker	4 (3)
Student	1 (1)
Unemployed	10 (7)
Retired	15 (11)
Declined	6 (4)
Marital status	
Never married	22 (16)
Domestic partnership	11 (8)
Married	55 (41)
Separated	7 (5)
Divorced	5 (4)
Relation to survivor	
Spouse/partner	43 (32)
Child	23 (17)
Parent	17 (13)
Sibling	12 (9)
Extended family or other	5 (4)
Witnessed the cardiac arrest	24 (18)
Psychological distress (PCL-5 total score), mean ± SD	22 ± 18

Note. Values are % (*n*) unless otherwise reported. GED = Graduate Education Diploma. PCL-5 = Post-Traumatic Stress Disorder Checklist for DSM-5.

**Table 3:** Associations between psychological distress of close family members and predictors at 1 Month after cardiac arrest.

Covariate	Univariate Coefficient (B)	95% CI	*p* Value	Standardized Beta (β)	Model 1 Coefficient (B)	95% CI	*p* Value	Standardized Beta (β)	Model 2 Coefficient (B)	95% CI	*p* Value	Standardized Beta (β)	Model 3 Coefficient (B)	95% CI	*p* Value	Standardized Beta (β)
PCL-5 total score (Survivor)	0.4	0.2, 0.6	<0.01	0.4	0.4	0.1, 0.6	<0.01	0.4	0.3	0.1, 0.6	0.02	0.3	0.3	0.1, 0.6	0.02	0.3
PSMS (Survivor)	−0.2	−2.1, 1.7	0.8	0.0	−0.1	−2.0, 1.7	0.9	0.0	0.3	−1.6, 2.3	0.7	0.0	−0.1	−2.1, 2.0	0.9	0.0
TICS-M (Survivor)	−0.3	−0.9, 0.3	0.3	−0.1	−0.2	−0.8, 0.4	0.5	−0.1	−0.2	−0.9, 0.4	0.5	−0.1	−0.2	−0.8, 0.5	0.5	−0.1
Age (Family)	−0.3	−0.6, 0.0	0.1	−0.2					−0.1	−0.4, 0.2	0.6	−0.1	−0.1	−0.4, 0.2	0.6	−0.1
Female sex (Family)	−3.5	−12.8, 5.8	0.5	−0.1					−5.1	−14.4, 4.2	0.3	−0.1	−6.3	−15.8, 3.2	0.2	−0.2
High Education status (Family)	−7.0	−15.5, 1.4	0.1	−0.2					−8.1	−16.9, 0.8	0.1	−0.2	−8.9	−17.8, 0.0	0.1	−0.3
Spouse/Partner (Family)	−1.3	−9.8, 7.2	0.8	0.0					−1.9	−10.5, 6.6	0.7	−0.1	−1.9	−10.4, 6.7	0.7	−0.1
Hospital Length of Stay	−0.1	−0.2, 0.1	0.37	−0.1									−0.1	−0.2, 0.0	0.2	−0.2

Note. PCL-5 = Post-traumatic stress disorder checklist for the Diagnostic and Statistical Manual 5. PSMS = physical self-maintenance scale. TICS-M = modified telephone interview for cognitive status.

**Table 4:** Spearman’s correlation matrix for family member and survivor PCL-5 total and subscales.

	Family PCL-5 Total Score	Family—Intrusion	Family—Avoidance	Family—Negative Alteration	Family—Hyperarousal	Survivor PCL-5 Total Score	Survivor—Intrusion	Survivor—Avoidance	Survivor—Negative Alteration	Survivor—Hyperarousal
Family PCL-5 Total Score	1									
Family—Intrusion	0.76 ***	1								
Family—Avoidance	0.62 ***	0.67 ***	1							
Family—Negative Alteration	0.67 ***	0.72 ***	0.69 ***	1						
Family—Hyperarousal	0.68 ***	0.74 ***	0.71 ***	0.82 ***	1					
Survivor PCL-5 Total Score	0.36 **	0.35 **	0.36 **	0.44 ***	0.29 *	1				
Survivor—Intrusion	0.30 **	0.35 **	0.35 **	0.41 ***	0.25 *	0.88 ***	1			
Survivor—Avoidance	0.29 *	0.36 **	0.37 **	0.46 ***	0.29 *	0.77 ***	0.66 ***	1		
Survivor—Negative Alteration	0.49 ***	0.34 **	0.30 *	0.44 ***	0.31 **	0.90 ***	0.75 ***	0.61 ***	1	
Survivor—Hyperarousal	0.30 *	0.30 *	0.35 **	0.31 **	0.23 *	0.87 ***	0.65 ***	0.60 ***	0.75 ***	1

Note. PCL-5 = PTSS, PTSD Checklist—PCL 5. * *p* < 0.05. ** *p* < 0.01. *** *p* < 0.001.

## Data Availability

The datasets presented in this article are not readily available because the data are part of an ongoing study. Requests to access the datasets should be directed to sa2512@columbia.edu.

## Supplementary Materials

The following supporting information can be downloaded at: https://www.mdpi.com/article/10.3390/jcm13175266/s1, Figure S1: Kernel Density Estimate of Post-Traumatic Stress Scores in Close Family Members One Month Post-Arrest. The plot shows the distribution of PCL-5 scores with KDE smoothing applied. The *x*-axis represents PCL-5 score values, and the *y*-axis indicates density; Figure S2: CONSORT diagram showing the flow of participants (survivors and close family members); Table S1: Comparing Family member attributes and survivor characteristics in close family members with or without psychological distress at one-month after cardiac arrest; Table S2: Univariate Associations of Survivor and Family Characteristics with Psychological Distress of Close Family Members at one-month After Cardiac Arrest; Table S3: Comparing family member characteristics of those completing one-month assessments versus lost to follow-up.
