# Supplementary material for: Associations between Post-Intensive Care Syndrome Domains in Cardiac Arrest Survivors and Their Families One Month Post-Event
Source: J Clin Med. 2024 Sep 5;13(17):5266. doi: 10.3390/jcm13175266 (PMC11396683; doi:10.3390/jcm13175266)
Supplement: Supplementary file 1 [file jcm-13-05266-s001.zip › jcm-3191968-supplementary.pdf]

SUPPLEMENTAL METHODS

1. STROBE Statement—Checklist of items that should be included in reports of ***cohort studies***

	Item No	Recommendation	Page No
**Title and abstract**	1	(*a*) Indicate the study's design with a commonly used term in the title or the abstract<br>(*b*) Provide in the abstract an informative and balanced summary of what was done and what was found	1<br>1
Background/rationale	2	Explain the scientific background and rationale for the investigation being reported	1-2
Objectives	3	State specific objectives, including any prespecified hypotheses	2
Study design	4	Present key elements of study design early in the paper	2-3
Setting	5	Describe the setting, locations, and relevant dates, including periods of recruitment, exposure, follow-up, and data collection	2
Participants	6	Give the eligibility criteria, and the sources and methods of selection of participants. Describe methods of follow-up	3
Variables	7	Clearly define all outcomes, exposures, predictors, potential confounders, and effect modifiers. Give diagnostic criteria, if applicable	3-5
Data sources/ measurement	8*	For each variable of interest, give sources of data and details of methods of assessment (measurement). Describe comparability of assessment methods if there is more than one group	3-5
Bias	9	Describe any efforts to address potential sources of bias	16-17
Study size	10	Explain how the study size was arrived at	5
Quantitative variables	11	Explain how quantitative variables were handled in the analyses. If applicable, describe which groupings were chosen and why	5
Statistical methods	12	(*a*) Describe all statistical methods, including those used to control for confounding<br>(*b*) Describe any methods used to examine subgroups and interactions<br>(*c*) Explain how missing data were addressed<br>(*d*) If applicable, explain how loss to follow-up was addressed<br>(*e*) Describe any sensitivity analyses	4-5<br>5<br>N/A<br>N/A<br>N/A
**Results**			
Participants	13*	(a) Report numbers of individuals at each stage of study—eg numbers potentially eligible, examined for eligibility, confirmed eligible, included in the study, completing follow-up, and analysed	5

		(b) Give reasons for non-participation at each stage	5
		(c) Consider use of a flow diagram	5
Descriptive data	14*	(a) Give characteristics of study participants (eg demographic, clinical, social) and information on exposures and potential confounders	5-8
		(b) Indicate number of participants with missing data for each variable of interest	N/A
		(c) Summarise follow-up time (eg, average and total amount)	4
Outcome data	15*	Report numbers of outcome events or summary measures over time	8-9
Main results	16	(*a*) Give unadjusted estimates and, if applicable, confounder-adjusted estimates and their precision (eg, 95% confidence interval). Make clear which confounders were adjusted for and why they were included	10
		(*b*) Report category boundaries when continuous variables were categorized	10
Other analyses	17	Report other analyses done—eg analyses of subgroups and interactions, and sensitivity analyses	10-14

Key results	18	Summarise key results with reference to study objectives	14-16
Limitations	19	Discuss limitations of the study, taking into account sources of potential bias or imprecision. Discuss both direction and magnitude of any potential bias	16
Interpretation	20	Give a cautious overall interpretation of results considering objectives, limitations, multiplicity of analyses, results from similar studies, and other relevant evidence	14-17
Generalisability	21	Discuss the generalisability (external validity) of the study results	16-17

Funding	22	Give the source of funding and the role of the funders for the present study and, if applicable, for the original study on which the present article is based	17

Supplementary Figure S1: Kernel Density Estimate of Post-Traumatic Stress Scores in Close Family Members One Month Post-Arrest. The plot shows the distribution of PCL-5 scores with KDE smoothing applied. The x-axis represents PCL-5 score values, and the y-axis indicates density.

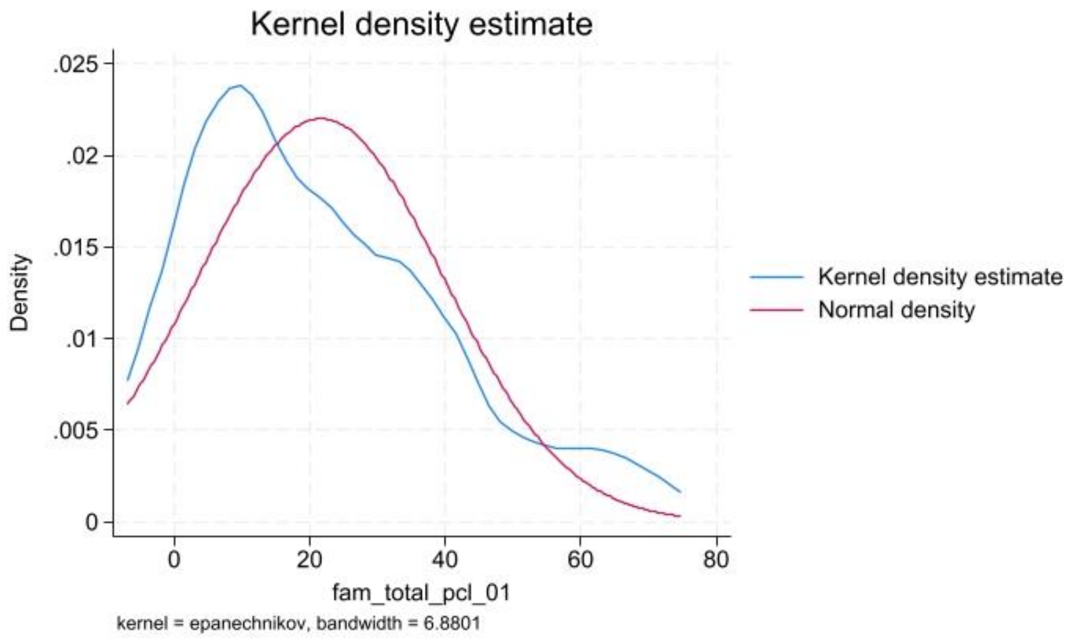

Supplementary Figure S2: Consort diagram showing the flow of participants (survivors and close family members)

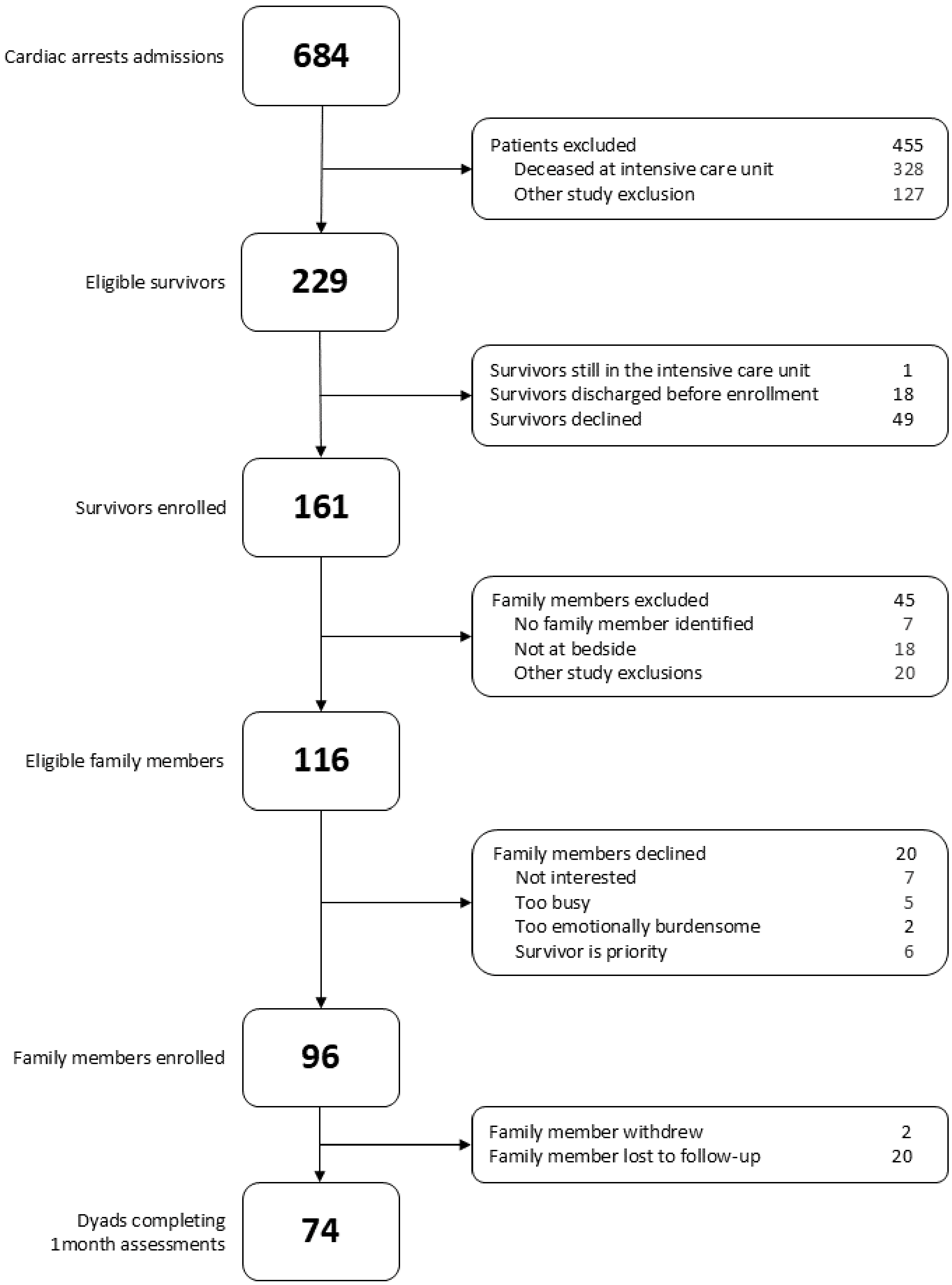

Supplementary Table S1: Comparing Family member attributes and survivor characteristics in close family members with or without psychological distress at one-month after cardiac arrest

	**Psychological Distress, 30% (N=22)**	**No Psychological Distress, 70% (N=52)**	*p-value*
**Family Member Attributes**			
Age, years (Mean ± Standard Deviation)	52 ± 14	53 ± 13	0.8
Female Sex	82 (18)	67 (35)	0.3
Race/Ethnicity			
Non-Hispanic White	5 (1)	44 (23)	
Hispanic/Latino	77 (17)	39 (20)	
Black	5 (1)	13 (7)	
Other	13 (3)	4 (2)	**<0.01**
Language			
English	77 (17)	87 (45)	0.3
Spanish	23 (5)	13 (7)	
Marital status			
Never married	14 (3)	23 (12)	
Domestic partnership	22 (5)	6 (3)	
Married	41 (9)	63 (32)	
Separated	14 (3)	4 (2)	
Divorced	9 (2)	4 (2)	0.1
Relationship with the patient			
Partner/spouse	45 (10)	42 (22)	
Child	27 (6)	19 (10)	
Parent	5 (1)	23 (12)	
Sibling	23 (5)	8 (4)	
Extended family or other	0	8 (4)	0.1
Educational attainment			
Less than college degree	67 (14)	43 (22)	
College degree or higher education	33 (7)	57 (29)	0.1
Occupational status			
Employed, full-time	38 (8)	44 (23)	
Employed, part-time	9 (2)	13 (7)	
Disabled, permanent or temporary	4 (1)	6 (3)	
Temporarily laid off, sick leave, or maternity leave	0	4 (2)	
Keeping house	4 (1)	4 (2)	
Student	0	2 (1)	
Unemployed, looking for work	9 (2)	10 (5)	
Retired	19 (4)	13 (7)	
Declined	19 (4)	4 (2)	0.8

Witnessed the Cardiac Arrest	32 (7)	20 (10)	0.4
**Survivor Attributes**			
Age, years (Mean ± Standard Deviation)	58 ± 13	55 ± 16	0.5
Female sex	32 (7)	42 (22)	0.4
Sexuality			
Heterosexual	100 (22)	98 (51)	
Bisexual	0	2 (1)	1.0
Race/ Ethnicity			
Non-Hispanic white	41 (90)	48 (25)	
Blacks	9 (2)	19 (10)	
Asians	5 (1)	0	
Hispanic/Latino	46 (10)	33 (17)	0.2
Education			
High school diploma/ GED or less	64 (14)	37 (19)	
Trade school/ some college or more	36 (8)	63 (33)	**0.04**
Income			
Less than $59,000	54 (12)	44 (23)	
$60,000-$89,000	9 (2)	8 (4)	
Greater than $90,000	23 (5)	29 (15)	
Unknown	14 (3)	19 (10)	0.9
Language			
English	86 (19)	81 (42)	
Spanish	14 (3)	19 (10)	0.7
How patient learned about Cardiac Arrest			
Clinical staff	18 (4)	46 (24)	
Research staff	14 (3)	2 (1)	
Family member	50 (11)	35 (18)	
Self	18 (4)	17 (9)	0.04
Remember the Cardiac Arrest	14 (3)	15 (8)	1.0
Psychological distress (PCL-5)	30 ± 14	21 ± 17	**0.01**
Physical independence (PSMS)	3 ± 2	3 ± 2	0.9
Cognitive function (TICS-M)	30 ± 7	32 ± 7	0.2
Cardiac Arrest site			
Out-of-Hospital	32 (7)	25 (13)	
In-Hospital	68 (15)	75 (39)	0.6
Initial Rhythm			

Unknown	5 (1)	10 (5)	
VT†/VF‡/AED Advised Shock	50 (11)	25 (13)	
Asystole/PEA §	45 (10)	65 (34)	0.1
Length of hospitalization	42 ± 47	38 ± 37	0.4
Time to ROSC*	10 ± 11	11± 17	0.9
Discharge disposition after Cardiac Arrest			
Home	82 (18)	45 (23)	
Facility	18 (4)	55 (28)	**<0.01**
Charlson Comorbidity Index	3 ± 2	3 ± 2	0.8
Health insurance status			
Uninsured	0	2 (1)	
Medicaid	41 (9)	46 (23)	
Medicare	32 (7)	26 (13)	
Private	27 (6)	26 (13)	0.9
Cause of Cardiac Arrest			
Unknown	9 (2)	0	
Cardiac Etiology	45 (10)	46 (24)	
Respiratory Etiology	18 (4)	27 (14)	
Circulatory Etiology	28 (6)	21 (11)	
Other	0	6 (3)	0.2

† VT = Ventricular Tachycardia
‡ VF = Ventricular Fibrillation
§ PEA = Pulseless Electrical Activity
* ROSC = Return to spontaneous circulation

Supplementary Table S2: Univariate Associations of Survivor and Family Characteristics with Psychological Distress of Close Family Members at one-month After Cardiac Arrest

**Covariates**	**Coefficients**	**95% CI**	***p* value**
**Survivor Characteristics**			
Age, years	0.1	-0.2, 0.4	0.4
Female vs Male sex	0.1	-8.4, 8.7	0.9
Race/ethnicity			
Non-Hispanic White	1		
Black	-8.1	-20, 3.8	0.2
Hispanic/Latino	1.5	-7.6, 10.6	0.7
Trade school/some college or more	-8.7	-16.9, -0.5	0.0
Income less than $59,999 vs $60,000 or above	4.5	-3.9, 12.8	0.3
Spanish Language	-2.6	-13.6, 8.4	0.6
Cardiac Arrest site (in-hospital vs out-of-hospital cardiac arrest)	-6.2	-15.6, 3.2	0.2
Initial Rhythm (Non-Shockable vs Shockable)	-1.8	-8.4, 4.7	0.6
Return of Spontaneous Circulation	-0.1	-0.4, 0.2	0.6
Charlson Comorbidity Index	-0.1	-1.9, 1.7	0.9
Poor Insurance Status (no insurance or Medicaid vs others)	-0.4	-8.9, 8.2	0.9
Poor Functional Status (Modified Rankin Scale Score>2)	-6.7	-15.4, 1.9	0.1
Discharge Disposition (Inpatient Rehab Facility vs Home)	-13.8	-21.7, -5.8	**<0.01**
**Family Member Characteristics**			
Race/ethnicity			
Non-Hispanic White	1		
Black	-0.1	-13, 13	0.9
Hispanic/Latino	16	7.4, - 24.4	**<0.01**
Language Fluency (Spanish vs English)	4.6	-6.8, 16.0	0.4
Relationship status (Spouse/Partner vs not)	-1.3	-9.8, 7.2	0.8
Employed full-time vs others	-2.1	-10.7, 6.4	0.6
Witnessed the Cardiac Arrest	6.2	-4, 16	0.2

Supplementary Table S3: Comparing family member characteristics of those completing one-month assessments versus lost to follow-up

	**Participants Analyzed (N= 74) % (n)**	**Participants Lost to follow-up (N= 22) % (n)**	***p-value***
**Family Member Characteristics**			
Age, years, Mean ± SD	52 ± 14	50 ± 14	0.5
Female Sex	72 (54)	68 (15)	0.7
Race/ethnicity			
Non-Hispanic White	32 (24)	50 (11)	
Black	11 (8)	14 (3)	
Hispanic/Latino	51 (38)	36 (8)	
Other	6 (5)	0 (0)	0.2
Language			
English	84 (63)	86 (19)	
Spanish	16 (12)	14 (3)	0.8
Educational attainment			
8th grade or less	1 (1)	0 (0)	
Some high school	8 (6)	5 (1)	
High school diploma or GED	10 (7)	9 (2)	
Trade school/vocational school	8 (6)	5 (1)	
Some college, no degree	22 (16)	9 (2)	
College degree	30 (22)	27 (6)	
Some graduate school, no degree	4 (3)	13 (3)	
Graduate degree	17 (12)	32 (7)	0.1
Employment status			
Employed, full-time	43 (32)	45 (10)	
Employed, part-time	12 (9)	9 (2)	
Disabled, permanently or temporarily	6 (4)	5 (1)	
Temporarily laid off, sick or maternity leave	3 (2)	9 (2)	
Homemaker	4 (3)	5 (1)	
Student	1 (1)	0 (0)	
Unemployed	10 (7)	9 (2)	
Retired	15 (11)	18 (4)	
Declined	6 (4)	0 (0)	0.8
Marital status			
Never married	22 (16)	18 (4)	
Domestic partnership	11 (8)	14 (3)	
Married	55 (41)	55 (12)	
Separated	7 (5)	9 (2)	
Divorced	5 (4)	4 (1)	0.7
Relation to survivor			

Spouse/partner	43 (32)	36 (8)	
Child	23 (17)	23 (5)	
Parent	17 (13)	23 (5)	
Sibling	12 (9)	5 (1)	
Extended family or other	5 (4)	13 (3)	0.6
Witnessed the cardiac arrest	26 (19)	14 (3)	0.2
**Survivor Characteristics**			
Age, years, Mean ± SD	56 ± 16	54 ± 20	0.6
Female Sex	39 (29)	36 (8)	0.8
Insurance			
Uninsured	1 (1)	0 (0)	
Insured (Medicaid)	43 (32)	27 (6)	
Insured (Medicare)	28 (20)	41 (9)	
Insured (Private)	28 (20)	32 (7)	0.1
Cardiac Arrest site			
Out-of-Hospital	27 (20)	9 (2)	
In-hospital	73 (55)	91 (20)	0.1
ICU length of stay, days, Median (Interquartile Range)	13 (8 – 26)	14 (7 – 20)	0.7
Disposition			
Home	57 (41)	59 (13)	
Facility	43 (31)	41 (9)	0.9
Poor functional status, Modified Rankin Scale Score>2	66 (49)	68 (15)	0.8
